# Differing Transcriptomic Responses in High Titer versus Low Titer *Aedes aegypti* Mosquitoes after Oral Infection with Sindbis Virus

**DOI:** 10.3390/v16091487

**Published:** 2024-09-19

**Authors:** Peter Hodoameda, Robert E. Ditter, Scott R. Santos, Rollie J. Clem

**Affiliations:** 1 Division of Biology, Kansas State University, Manhattan, KS 66506, USA; hodoamed@email.unc.edu; 2 Department of Biological Sciences, State University of New York at Buffalo, Buffalo, NY 14261, USA; rditter@buffalo.edu

**Keywords:** arbovirus, vector competence, *Aedes aegypti*, mosquito, Sindbis virus

## Abstract

Oral infection of mosquitoes by arboviruses often results in a large degree of variation in the amount of infectious virus between individual mosquitoes, even when the mosquitoes are from inbred laboratory strains. This variability in arbovirus load has been shown to affect virus transmissibility. Previously, our group described population genetic and specific infectivity differences between the virus populations found in high and low titer *Aedes aegypti* mosquitoes that had been orally infected with Sindbis virus (SINV). In this study, we sought to investigate whether there were also differences in transcriptomic response between these high and low titer mosquitoes. Results from the transcriptomic data analysis showed that more genes involved in antiviral activity, endopeptidase activity, and methyltransferase activity were upregulated in low titer mosquitoes than in high titer mosquitoes, relative to blood-fed controls. Meanwhile, genes involved in ion transport, energy metabolism, acetylation, glycosylation, lipid metabolism, and transport tended to be upregulated in high titer mosquitoes more than in low titer mosquitoes, relative to blood-fed mosquitoes. Overall, genes involved in antiviral activities tended to be upregulated in low titer mosquitoes while genes involved in proviral activities were mostly upregulated in high titer mosquitoes. This study has identified a number of candidate mosquito genes that are putatively associated with SINV titer variability after oral infection of *Ae. aegypti*, and these can now be investigated in order to ascertain their roles in virus replication and their contributions to determining vector competence.

## 1. Introduction

Mosquito vector competence is the intrinsic ability of a mosquito to acquire, maintain, and transmit arboviruses, and a mosquito’s genetic makeup contributes significantly to its vector competence [1,2]. Among medically important arbovirus vectors, the yellow fever mosquito *Aedes aegypti* is uniquely significant due to its competence for transmitting several arboviruses of public health importance, including dengue virus (DENV), Zika virus (ZIKV), yellow fever virus (YFV), and chikungunya virus (CHIKV) [3,4].

When a mosquito ingests blood containing an arbovirus, a wide range of mechanisms and barriers are present to attempt to counteract the infection [4]. Anatomical barriers to infection or transmission include the midgut infection and escape barriers, as well as the salivary gland infection and escape barriers [5]. In addition to these physical barriers, the arbovirus–mosquito interaction also leads to changes in the expression of numerous vector genes involved in immune pathways, metabolism, ion and solute transportation, energy production, and many other processes [6,7,8,9].

Whole genome-based transcriptomic analysis has been used extensively to study the genetic interactions between *Ae. aegypti* and arboviruses, and these studies have revealed the vector response during arbovirus infection, dissemination, and transmission in the mosquito [10,11,12,13]. Many candidate genes in *Ae. aegypti* have been shown to be differentially regulated following infection with different arboviruses [14,15,16]. For example, a study using *Ae. aegypti* (Rockefeller strain) infected with one of three different flaviviruses, namely DENV-2, YFV, or West Nile virus (WNV), identified 35 differentially expressed (DE) genes that were shared in mosquitoes infected by all three viruses. On the other hand, the authors also identified more than 400 other genes whose expression was differentially regulated after infection with only one or two of the different viruses [8], indicating the complexity of the responses to different arboviruses. Understanding mosquito transcriptomic responses to arboviruses is a vital tool in our understanding of mosquito vector competence [10,17] and contributes important new knowledge to the development of novel vector control strategies [4]. Although many previous studies have identified a number of genes whose expression is differentially regulated after arbovirus infection, with one exception, they have focused on susceptibility or refractoriness of a mosquito population as a whole, and not on arbovirus titer variability between individual mosquitoes [18,19,20]. To our knowledge, the lone exception to this is a study by Raquin et al., who performed RNAseq on individual field-caught *Ae. aegypti* mosquitoes and used a unique bioinformatic approach that identified several candidate genes associated with differences in DENV titers. However, the wild-caught mosquitoes used by Raquin et al. would be expected to be relatively genetically diverse compared to inbred laboratory strains [19].

Previously, our group has shown that there is enormous variation (at least 10,000-fold) in virus titers between individual mosquitoes after oral infection of inbred laboratory strains of *Ae. aegypti* with SINV [21,22]. Since these laboratory strains would be expected to be less genetically diverse than wild populations, this raises the question of what is responsible for this huge degree of variation in individual titers. Our previous results indicated that at least part of this variation can be explained by differences in the virus populations found in low titer versus high titer mosquitoes [22]. Viruses in low and high titer mosquitoes differed in their population structures, with low titer viruses exhibiting stronger reductions in genetic complexity and diversity following dissemination from the midgut, accompanied by stronger negative selection than in high titer viruses. In addition, despite their dramatic differences in titers of infectious virus, low and high titer mosquitoes contained similar amounts of viral genomes, indicating that viruses from high titer mosquitoes had higher specific infectivity (the ratio of infectious units to viral genomes) than those from low titer mosquitoes [22]. Additionally, we showed that specific infectivity was increased by antibiotic treatment, a known modifier of mosquito antiviral immunity, suggesting that the mosquito immune response can affect the efficiency of SINV genome encapsidation and thus the virus titers.

To further investigate the role of mosquito responses in causing variability in SINV titers after oral infection, here, we have analyzed RNAseq data obtained from orally infected mosquitoes that contained either high or low titers of infectious SINV and identified differentially expressed genes (DEGs) in the midguts and carcasses of these mosquitoes relative to blood-fed mosquitoes (mosquitoes that ingested a blood meal without a virus). Overall, our results reveal that numerous genes that are predicted to enhance virus replication (proviral genes) are upregulated in high titer mosquitoes relative to blood fed ones, while many genes with predicted antiviral activity are upregulated in low titer mosquitoes when compared to blood fed ones. These results further define the genetic factors that contribute to titer variation during arbovirus infection in mosquitoes and identify a number of candidate genes that can be tested in the future for their effects on arbovirus replication in mosquitoes.

## 2. Materials and Methods

### 2.1. Acquisition of Sequence Data

The RNAseq data used in this study were derived from the same data that were previously used to characterize the SINV populations in midgut and carcass samples from 6 low titer and 6 high titer *Ae. aegypti* (Orlando) mosquitoes [22]. In addition, sequences from 3 blood-fed control mosquitoes that were collected and processed at the same time and using the same methodology as the data described previously [22] were also used. The raw sequence data are available in the Sequence Read Archive (https://www.ncbi.nlm.nih.gov/sra) (accessed on 17 September 2024) under BioProject ID PRJNA1000578.

### 2.2. RNAseq Data Analysis

*Ae. aegypti* RNA sequences were aligned to the reference genome AaegL5.3 obtained from VectorBase (httpes://www.vectorbase.org/organisms/aedes-aegypti) with Bowtie 2 2.3.4.1 using the “very-sensitive” option [18,19,20]. All reads that failed to align to AaegLv5.3 with a minimum of 10× coverage were discarded, and the remaining sequences were converted to bam format and sorted using Samtools v1.9 [23]. The reference genome AaegLv5.3 was prepared to quantify transcript abundance and generate count matrices using RSEM 1.2.20 [24]. Differential expression (DE) levels of transcribed genes for each sample were identified using RSEM 1.2.20, and the results were filtered for false discovery rate (FDR) with the parameters logFC ≥ 0.05 and *p* < 0.05 [24]. Analyses were conducted on a high-performance computation cluster at the Center for Computational Research at the University at Buffalo.

To identify significant DEGs among sample groups, DE analysis was conducted with the package DESeq2 1.40.2 [25] in R v4.3.1 using RStudio 2023.06.1. DESeq2 was chosen because it has been shown to provide the most conservative results in reporting DEGs [26].

### 2.3. Principal Component Analyses (PCA) and Hierarchical Clustering Analyses (HCA)

Principal component analyses (PCA) were conducted on the basis of tissue type and infection level from DESeq2 results using the pcaExplorer 2.26.1 package in R [27,28]. The expression values of all significant DEGs were used to conduct hierarchical clustering analyses (HCA) as per [29].

### 2.4. Volcano Plot Analysis and Venn Diagrams

Volcano plot analysis was conducted with DESeq2 results (fold change ≥ 2 and FDR ≤ 0.05) using the EnhancedVolcano 1.18.0 package in R [30]. Venn diagrams of DEGs were generated using Venny 2.1.0.

### 2.5. GO Term Analysis and KEGG Pathway Analysis

Analyses of gene ontology and the KEGG pathway of DEGs were conducted using VectorBase and DAVID bioinformatics resources, respectively [31,32].

### 2.6. RNAseq Data Validation by RT-qPCR

Total RNA from mosquito midguts and carcasses was extracted using the PureLink RNA Mini Kit (Thermo Fisher Scientific, Waltham, MA, USA) according to the manufacturer’s instructions. A Power SYBR Green RNA-C_T_ 1-Step Kit (Thermo Fisher Scientific) was used for the RT-PCR reaction according to the manufacturer’s instructions. The primer sequences used are shown in Table S1. The cycling conditions for the reaction were the reverse transcription step at 48 °C for 30 min, then the enzyme activation step at 96 °C for 10 min, followed by 40 cycles of denaturation at 96 °C for 15 s and annealing/extension at 56 °C for 60 s. The RT-qPCR experiment for each sample was carried out in duplicate, and the results were averaged. Relative expression levels were calculated using the formula 2^−Δ*Ct*^, where Δ*Ct* is the difference in Ct values between the gene of interest and the housekeeping gene RPS7 (used as an internal control). Fold changes were calculated using the formula 2^−ΔΔ*Ct*^, where ΔΔ*Ct* is the difference in relative expression between the infected and the blood-fed control.

## 3. Results

### 3.1. Experimental Setup

**Figure 1:**
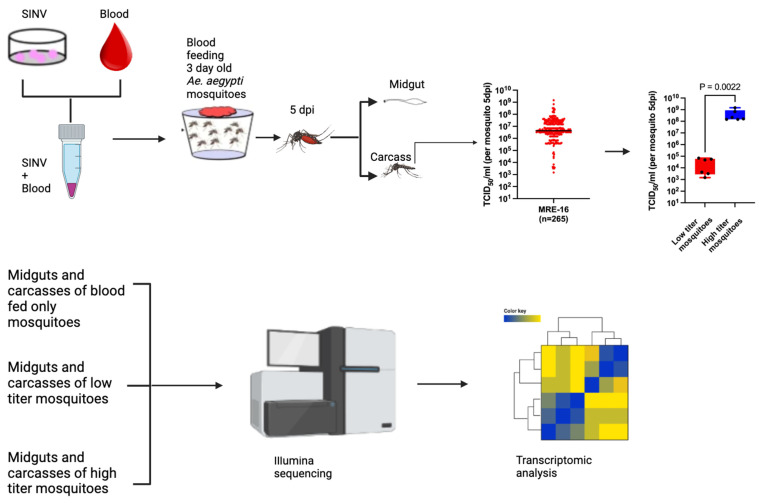
A schematic diagram showing the experimental setup used in this study. SINV was mixed with sheep blood and used to orally infect 3-day-old mosquitoes. At 5 days post blood meal, the mosquitoes were dissected into midguts and carcasses. Portions of the carcass samples were used to determine the titer of SINV in order to identify low titer and high titer mosquitoes. Poly(A)^+^ RNA was then isolated from the midguts and the remaining portions of the carcass samples from six low titer and six high titer samples, as well as from the midguts and carcasses of three control mosquitoes that were fed only blood, and the RNA was used to generate sequencing libraries that were then subjected to Illumina sequencing and analysis. The figure was generated using BioRender (https://www.biorender.com) (accessed on 17 September 2024). Some of the images in the figure were previously published [22].

To investigate the mosquito transcriptomic response contributing to differences in SINV load in *Ae. aegypti* after oral infection, we were able to utilize the same sequence data that we previously used to analyze viral sequences from high titer and low titer mosquitoes [22]. To briefly review how these data were generated, 3-day-old *Ae. aegypti* (Orlando) mosquitoes were orally infected using a Hemotek feeding system and sheep blood mixed with SINV derived from the infectious clone 5’dsMRE16ic (dose = 1.6 × 10^9^ TCID_50_/_mL_). At 5 days post blood meal, the midguts of the mosquitoes were dissected from the carcasses, and the SINV titer in the carcass was determined by TCID_50_ assay. Six low titer (≤6.8 × 10^4^ TCID_50_/mL) and six high titer (≥1.5 × 10^8^ TCID_50_/mL) mosquitoes were identified, and poly(A)^+^ RNA was isolated from the midguts and carcasses of these 12 mosquitoes, as well as from the midguts and carcasses of three control mosquitoes that had fed on the same batch of blood, but which did not contain the virus. Illumina sequencing was used to sequence the samples, and transcriptomic analysis was performed on the sequence data (Figure 1).

### 3.2. Principal Component Analysis and Hierarchy Cluster Analysis

**Figure 2:**
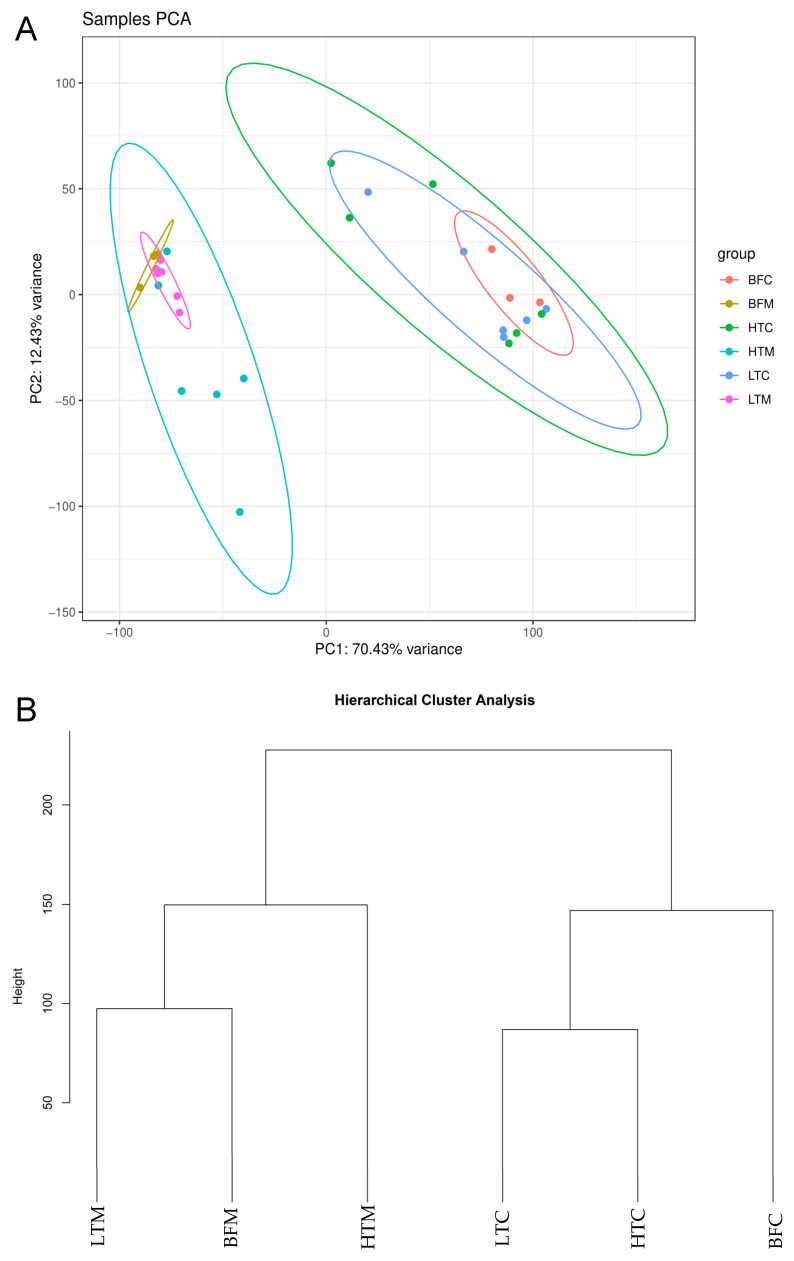
(**A**) Principal component analysis and (**B**) hierarchy cluster analysis of the transcriptomic data from six high titer mosquitoes, six low titer mosquitoes, and three blood fed mosquitoes. BFC, blood-fed carcass; BFM, blood-fed midgut; HTC, high titer carcass; HTM, high titer midgut; LTC, low titer carcass; LTM, low titer midgut. Each dot in (**A**) represents a single mosquito.

Total read counts from the sequenced samples ranged from 20.6 million to 37.7 million. After quality control (QC), read counts varied from 4 million to 22.1 million, and the percentage of reads that mapped to the *Ae. aegypti* genome ranged from 21–91%, with the average and median both being 68% (Table S2). In infected mosquitoes, varying proportions of the reads mapped to the polyadenylated SINV genome and were filtered out, thus reducing the proportion of reads mapping to *Ae. aegypti*; consistent with this, reads from uninfected blood-fed mosquito samples aligned to *Ae. aegypti* at rates of ≥89%, while the samples with the lowest alignment rates to *Ae. aegypti* were from high titer carcasses. Results from the principal component analysis (Figure 2A) and hierarchy cluster analysis (Figure 2B) showed that midgut samples and carcass samples clustered together in distinct groups, as would be expected since these are distinct sets of tissues with their associated transcriptional profiles. In the principal component analysis, all six of the low titer midgut samples clustered closely with the three blood-fed midgut samples, while four of the six high titer midgut samples were less closely associated (Figure 2A). This trend was also evident with the carcass samples; three of the six high titer carcasses were more divergent relative to blood-fed carcasses, while five of the six low titer carcasses were clustered tightly with the blood-fed ones. These results suggest a general trend towards increased variability in the gene expression patterns of high titer mosquitoes compared to low titer mosquitoes, both in midguts and carcasses.

### 3.3. Identification of DEGs

**Figure 3:**
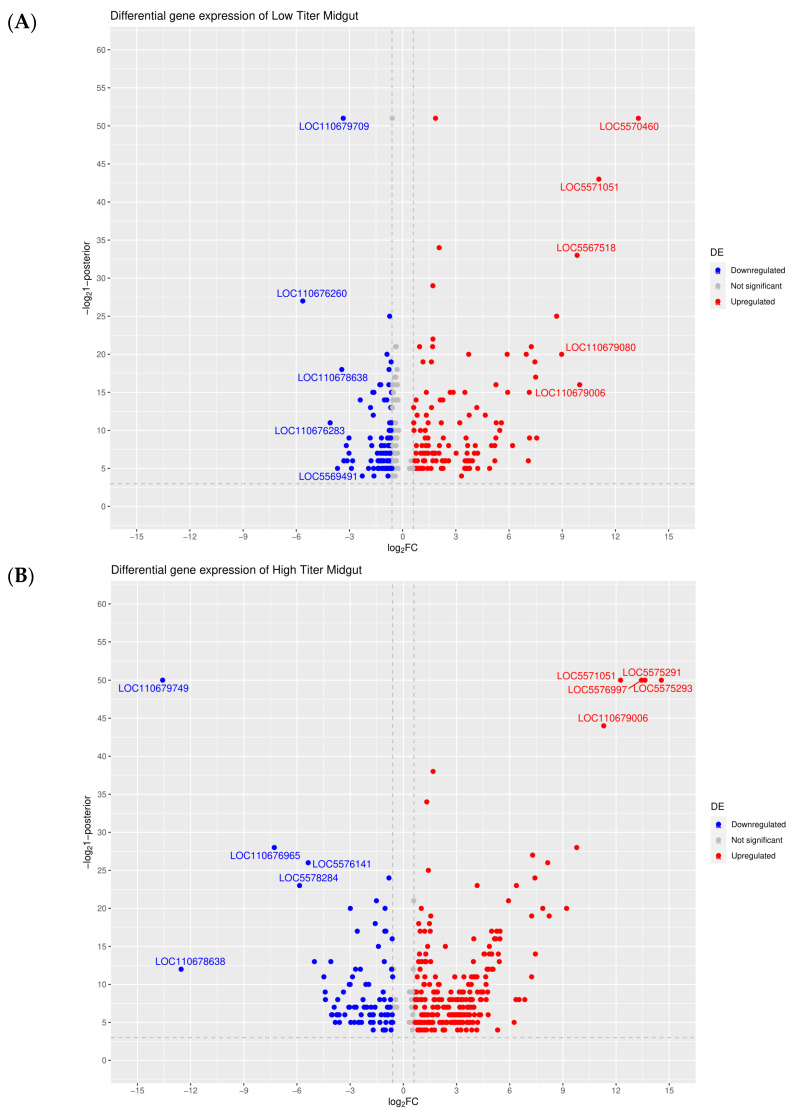

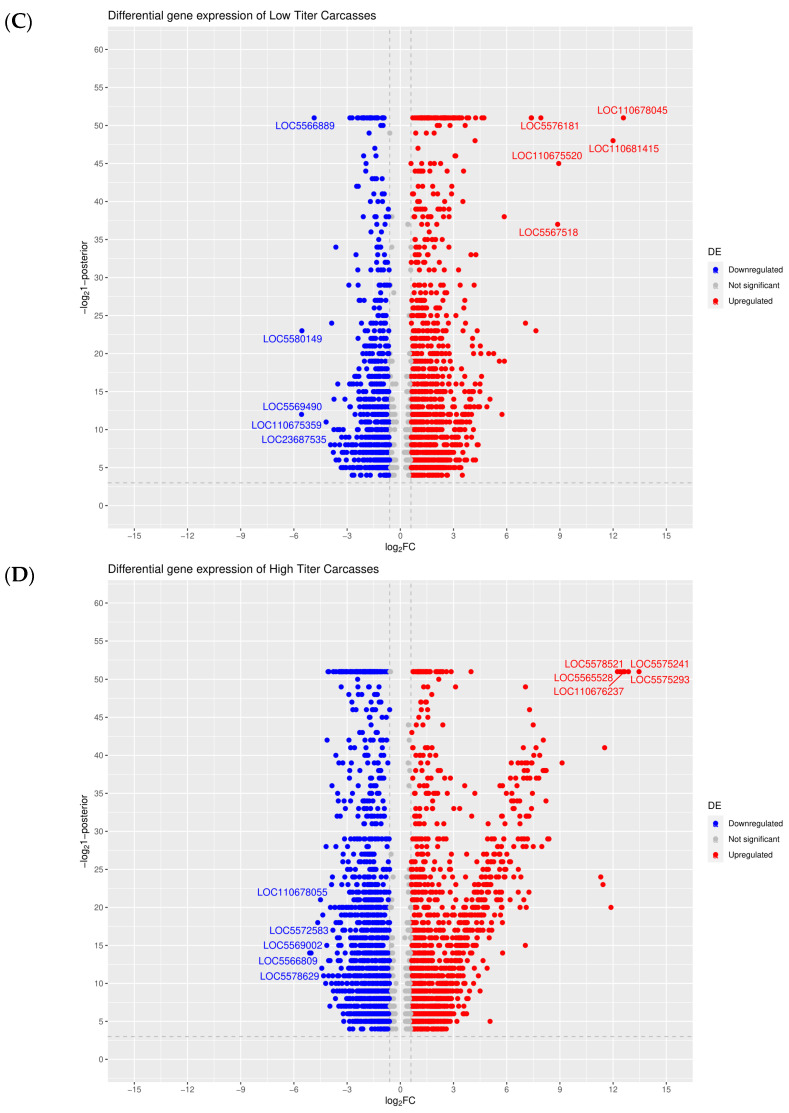
Volcano plot analysis of DEGs observed in (**A**) low titer midguts, (**B**) high titer midguts, (**C**) low titer carcasses, and (**D**) high titer carcasses (all relative to blood-fed ones). RefSeq identifiers are provided for the top five upregulated or downregulated DEGs in each category.

Analysis of the mRNA transcript expression levels showed that 52 genes had changes of two-fold or more in either direction in midguts of low titer mosquitoes when compared to blood-fed mosquitoes (Figure 3A), while 586 genes had changes of two-fold or more in either direction in midguts of high titer mosquitoes compared to blood-fed ones (Figure 3B). Overall higher numbers of DEGs were found in carcasses than in the midgut; the numbers of genes whose expression was altered two-fold or more in the carcasses of low titer mosquitoes and high titer mosquitoes (relative to blood fed) were 452 (Figure 3C) and 1209 (Figure 3D) genes, respectively. Lists of the DEGs with expression changes of ≥two-fold can be found in Supplementary File S1, while lists of the DEGs with expression changes of ≥10-fold are presented in Table 1.

To validate the RNAseq data, five of the DEGs exhibiting > 10-fold differences in expression relative to blood-fed ones were selected, and their transcript levels were analyzed by RT-qPCR and compared to the RNAseq read counts (Figure S1). A strong correlation (R^2^ = 0.8838) was observed between the RT-qPCR results and fold-expression changes (based on read counts) of these five genes, validating the RNAseq data.

### 3.4. Numbers of DEGs (Both Upregulated and Downregulated) That Were Unique Versus Shared between Samples

**Figure 4:**
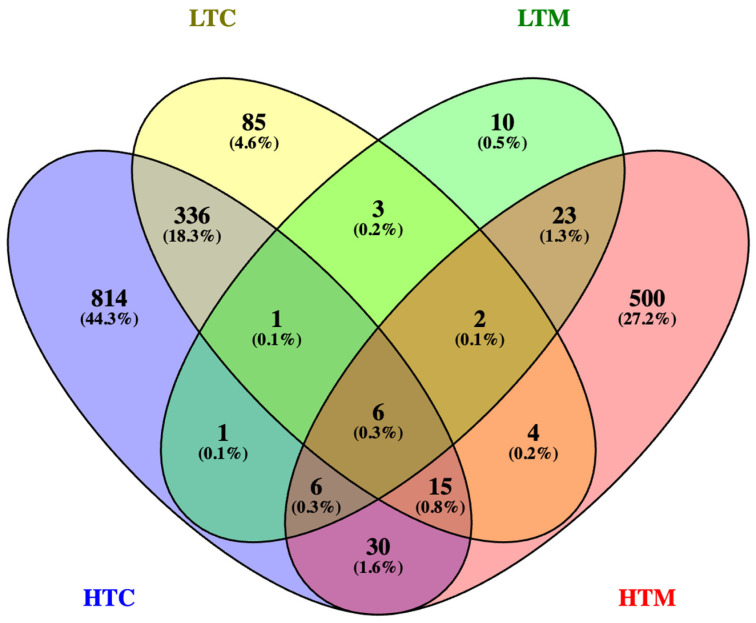
Venn diagram showing the numbers of DEGs with ≥two-fold expression difference (compared to blood-fed ones) that were unique or shared between low titer carcasses (LTC), low titer midguts (LTM), high titer carcasses (HTC), and high titer midguts (HTM).

Comparison of the transcriptomic data showed that 37 DEGs differing by ≥two-fold were shared between low titer midguts and high titer midguts, while 358 DEGs were shared between low titer carcasses and high titer carcasses (Figure 4). Furthermore, 12 DEGs were shared between low titer midguts and low titer carcasses, and 57 DEGs were shared between high titer midguts and high titer carcasses. Ten DEGs were unique to low titer midguts, and 500 were unique to high titer midguts, while 85 and 814 DEGs were unique to low titer carcasses and high titer carcasses, respectively. Only six DEGs were shared between all four types of samples, which were AAEL004369 (alpha-glucosidase), AAEL019786 (RYamide neuropeptide receptor), AAEL021204 (uncharacterized), LOC5576181 (peptidoglycan recognition protein), AAEL017563 (bifunctional endo-1,4-beta-xylanase XylA-like), and AAEL001364 (glucosyl/glucuronosyl transferase).

### 3.5. DEGs Predicted to Have Antiviral and Proviral Activity

**Figure 5:**
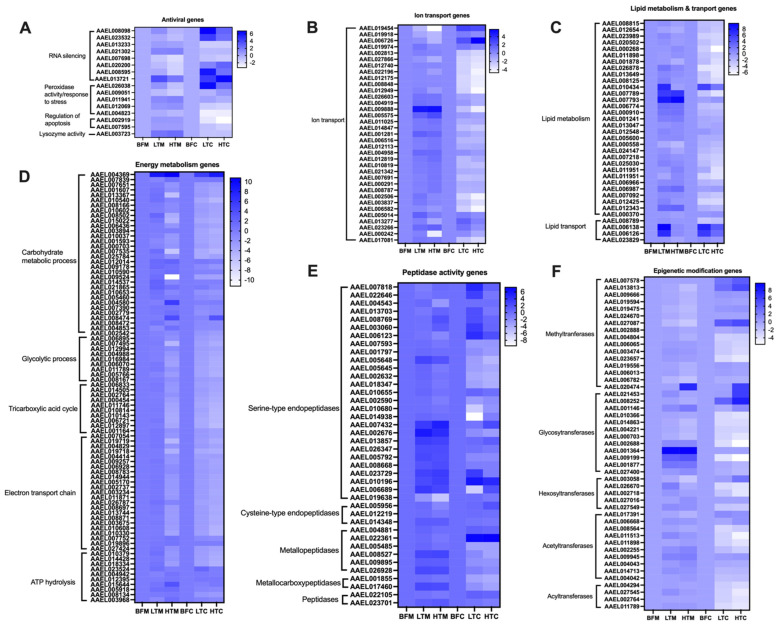
Heatmaps of DEGs involved in (**A**) antiviral activity, (**B**) ion transport, (**C**) lipid metabolism and transport, (**D**) energy metabolism, (**E**) peptidase activity, and (**F**) epigenetic modification. The averages of the gene matrix data were log_2_ transformed before they were used to generate the heatmaps.

Viral replication has been shown to be affected by numerous host cellular processes. In these data, we observed DEGs that are predicted to participate in processes such as antiviral response (16 genes), ion transport (35 genes), energy metabolism (84 genes), peptidase activity (40 genes), epigenetic modification of nucleic acid (47 genes), and lipid metabolism and transport (35 genes) (Figure 5). Of the 16 DEGs with predicted antiviral functions, eight are in RNA silencing pathways, five participate in peroxidase activity/response to stress, two regulate apoptosis, and one has predicted lysozyme activity (Figure 5A). A total of 35 DEGs are predicted to be involved in ion transport (Figure 5B), while 31 of the DEGs have predicted lipid metabolism function, and four are predicted to be involved in lipid transport (Figure 5C).

Virus replication requires energy from infected host cells; hence, differential expression of genes involved in energy metabolism may contribute to making energy available for viruses to use for replication in infected host cells. Our data revealed a number of DEGs predicted to be involved in energy metabolism. This included 31 genes involved in carbohydrate metabolism, nine involved in glycolysis, 10 involved in the tricarboxylic acid cycle, 22 involved in the electron transport chain, and 12 involved in ATP hydrolysis (Figure 5D). We also observed DEGs with predicted peptidase activity; of these, 27 are involved in serine-type endopeptidase function, three are cysteine-type endopeptidases, six have metallopeptidase activity, two have metallocarboxypeptidase function, and two have peptidase function (Figure 5E).

Epigenetic modification has been shown to affect virus infection and replication in host cells [33,34]. Of the DEGs with predicted epigenetic modification activity, 16 are methyltransferases, 12 are glycosyltransferases, five are hexosyltransferases, 10 are acetyltransferases, and four are acyltransferases (Figure 5F).

### 3.6. Gene Ontology (GO) Term Analysis Indicating the Predicted Molecular Functions and Biological Processes of DEGs

**Figure 6:**
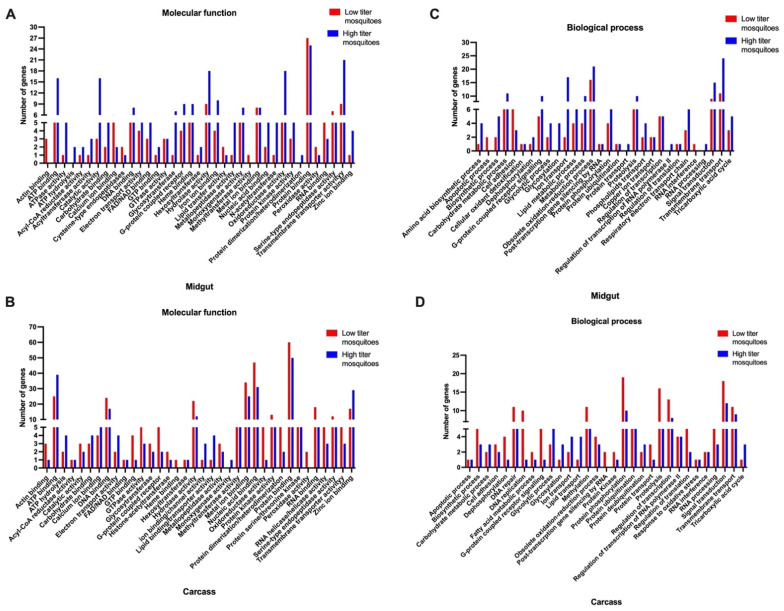
GO term analysis of (**A**) molecular functions of upregulated DEGs in midguts of low titer and high titer mosquitoes, (**B**) molecular functions of upregulated DEGs in carcasses of low titer and high titer mosquitoes, (**C**) biological processes of upregulated DEGs in midguts of low titer and high titer mosquitoes, and (**D**) biological processes of upregulated DEGs in carcasses of low titer and high titer mosquitoes. GO terms were analyzed using VectorBase.

GO term analysis of the molecular functions of genes upregulated in low titer and high titer mosquitoes showed that the products of these genes are predicted to have functions including acyl-coA reductases, ATP binding, ATP hydrolysis, iron ion binding, methyltransferase activity, calcium ion binding, hydrolase activity, nucleic acid binding, serine-type endopeptidase activity, oxidoreductase activity, protein kinase activity and zinc ion binding (Figure 6A,B). The GO term analysis for downregulated genes is shown in Figure S2.

GO term analysis of the biological processes of genes upregulated in midguts and carcasses of low titer and high titer mosquitoes included amino acid biosynthesis, apoptosis, cellular oxidant detoxification, lipid transport, G-protein coupled receptor activity, RNA processing, RNA interference, protein ubiquitination, and transmembrane transport (Figure 6C,D).

The GO term analysis of the cellular compartments where the products of DEGs are predicted to function is shown in Figure S3, while the proportions of characterized versus uncharacterized DEGs are shown in Figure S4.

### 3.7. KEGG Pathway Analysis of Enriched Pathways in Low Titer and High Titer Mosquitoes

**Figure 7:**
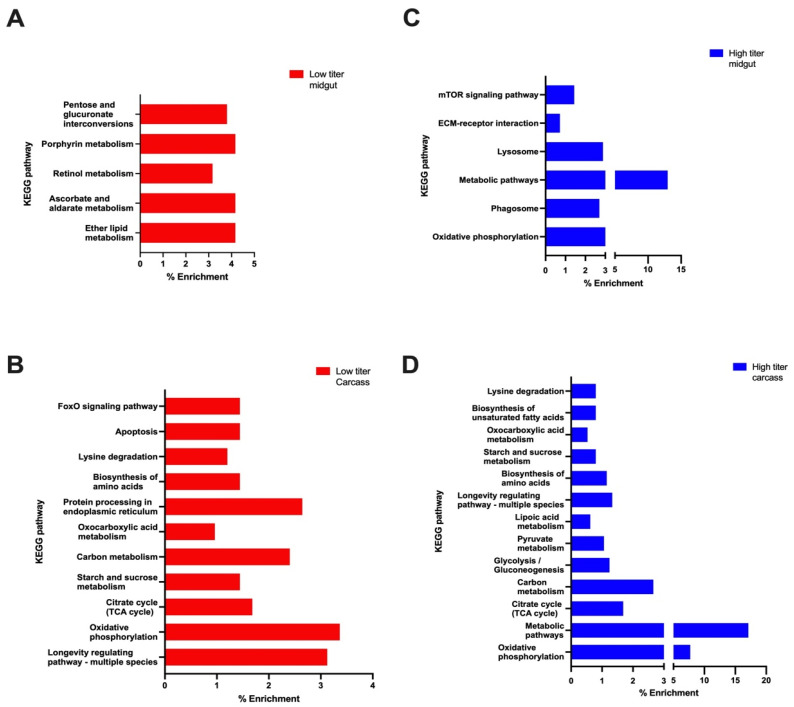
KEGG pathway analysis of (**A**) low titer midguts, (**B**) low titer carcasses, (**C**) high titer midguts, and (**D**) high titer carcasses.

The KEGG pathway analysis results showed that pathways enriched in midguts of low titer mosquitoes included pentose and glucuronate interconversions, porphyrin metabolism, retinol metabolism, ascorbate and aldarate metabolism, and ether lipid metabolism (Figure 7A). For the carcasses of low titer mosquitoes, the pathways enriched included FoxO signaling, apoptosis, lysine degradation, and protein processing in endoplasmic reticulum (Figure 7B). The pathways enriched in the midguts of high titer carcasses included mTOR signaling, ECM-receptor, lysosome, metabolism, and phagosome (Figure 7C). The enriched pathways in carcasses of high titer mosquitoes included biosynthesis of unsaturated fatty acid, oxocarboxylic acid metabolism, starch and sucrose metabolism, lipoic acid metabolism, pyruvate metabolism, and glycolysis (Figure 7D). Carbon metabolism, citrate metabolism (TCA cycle), amino acid biosynthesis, lysine degradation, longevity regulating pathway-multi species, and oxidative phosphorylation pathways were enriched in carcasses of both low titer and high titer mosquitoes (Figure 7C,D).

## 4. Discussion

A number of previous studies have described transcriptome changes in *Ae. aegypti* after oral infection with arboviruses such as CHIKV, DENV, WNV, ZIKV, and YFV [8,17,29,30,35,36]. However, these previous studies have mainly focused on overall susceptibility or refractoriness to arbovirus infection, with the exception of Raquin et al. [19], who examined variability in transcriptomic response among individual wild-caught mosquitoes infected with DENV. Here, we sought to investigate the contribution of host transcriptomic responses to SINV titer variability in an inbred strain of *Ae. aegypti*.

In this study, we compared the *Ae. aegypti* transcriptomic response in individual mosquitoes with differing SINV loads (high titer versus low titer) after oral infection. Overall, we found significant differences in the transcriptomes of these two groups of mosquitoes, compared to blood-fed controls. Principal component analysis indicated that there was more overall variation in the transcriptomes of high titer mosquitoes than of low titer mosquitoes, and this was reflected in higher numbers of DEGs in high titer samples. In addition, more DEGs (relative to blood-fed mosquitoes) were identified in carcasses than in midguts, presumably because carcasses contain a greater diversity of tissues and organs. Overall, the most prevalent differences in gene expression were in the functional categories of antiviral response, ion transport, lipid metabolism and transport, energy metabolism, peptidase activity, and epigenetic modification.

Several different types of antiviral mechanisms have been shown to be involved in defense against arbovirus infection in mosquitoes [6,37]. The antiviral genes differentially expressed in this study had functions including RNA silencing, peroxidase activity, regulation of apoptosis, and lysozyme activity. Notably, genes involved in the piRNA pathway, such as PIWI2, PIWI5, Maelstrom, and RNA helicases, were differentially expressed. As one might expect, antiviral genes tended to be more upregulated in both the midguts and carcasses of low titer mosquitoes (relative to blood-fed mosquitoes) than in high titer mosquitoes. This result is consistent with previous reports of higher expression of antiviral genes leading to decreases in arbovirus load in mosquitoes [6,37].

Mosquito peptidase activities are also important in determining virus infection [38]. Peptidases have been shown to be vital in mosquito immune regulation activities such as antimicrobial peptide synthesis, melanization, and coagulation of hemolymph [39,40,41]. Brackney and colleagues reported that serine-type endopeptidases like trypsins, which are important for blood digestion, may be associated with DENV replication in mosquito midgut [42]. Additionally, late phase trypsin (5GI) is associated with reduced DENV2 replication in the midgut [42]. This suggests that serine-type endopeptidases may function to reduce midgut infection in mosquitoes. In our study, we found that serine-type endopeptidases tended to be upregulated to a greater extent in low titer midguts and carcasses compared to blood-fed ones than in high titer midguts and carcasses.

Ion transport is vital in making ions available for the host cells to use for their cellular activities and may be important for arbovirus replication. A study by Sander et al. showed that SINV infection induced the upregulation of potassium-dependent sodium/calcium exchanger (NCKX), an ion transport gene which plays a significant role in Ca^2+^ transport [43]. Our results indicated that ion transport genes were more upregulated in the midguts and carcasses of high titer mosquitoes compared to blood-fed mosquitoes than in low titer mosquitoes compared to blood-fed ones.

Another important factor for arbovirus replication is lipid metabolism and transport. During infection of mosquitoes by arboviruses, there is an alteration of the lipid repertoire [44,45]. The alteration of lipid metabolic pathway(s) during arbovirus infection in the mosquito is important to achieve the optimal levels of different types of lipids required by arboviruses for replication, dissemination, and transmission. Additionally, higher lipid concentrations have been reported with increased arbovirus (e.g., DENV, ZIKV, and CHIKV) replication in mosquito cells [46]; hence, it is expected that a positive correlation between arbovirus load and the amounts of certain lipids produced in mosquito cells should exist. Results from our study showed that more genes involved in lipid metabolism and transport were upregulated in midguts and carcasses of high titer mosquitoes relative to blood-fed ones than in low titer mosquitoes compared to blood-fed ones [46].

The obligate intracellular nature of viruses means that arboviruses need to rely on the cell machinery for energy for replication. Like all viruses, arboviruses alter cellular energy metabolic pathways in ways that favor viral replication [47]. In one study, it was shown that pre-treatment of Vero cells with glycolysis inhibitors led to a significant reduction in Semliki Forest virus and SINV replication [47]. In our study, more genes involved in energy metabolism pathways including carbohydrate metabolism, glycolysis, and ATP hydrolysis were upregulated in midguts and carcasses of high titer mosquitoes compared to blood-fed mosquitoes than in low titer mosquitoes compared to blood-fed ones. Additionally, more genes in the electron transport chain and tricarboxylic acid cycles were downregulated in low titer carcasses compared to blood-fed ones than in high titer carcasses compared to blood fed ones.

Virus infectivity is affected by epigenetic modification, including post-transcriptional modification of RNA. RNA post-transcriptional modification is known to affect the replication of a number of viruses, in particular viruses with RNA genomes [48,49]. Depending on the type of post-transcriptional modification of viral RNA, virus replication and/or infectivity can be increased or decreased [50,51,52]. Results from our current study show that several genes whose products are involved in epigenetic modification of RNA were differentially regulated in the midguts and carcasses of high and low titer mosquitoes compared to blood-fed mosquitoes. Of particular interest was AAEL020474, a homolog of N^6^-methyltransferase that we observed to be highly upregulated in both midguts and carcasses of high titer mosquitoes. This is consistent with previous work showing that silencing an N^6^-methyltransferase in *Ae. aegypti* cells negatively impacted replication of DENV [52]. Additionally, genes involved in RNA acetylation and glycosylation, which usually have positive effects on viral replication, were mostly upregulated in the midguts and carcasses of high titer mosquitoes compared to blood fed.

In conclusion, our study has identified potentially important differences between the transcriptomes of high titer versus low titer mosquitoes after oral infection with SINV. Overall, the data reveal a general trend in which proviral genes are mostly upregulated in high titer mosquitoes while antiviral genes are mostly upregulated in low titer mosquitoes. This study has thus identified a number of candidate genes that can be tested in the future for their effects on SINV replication in *Ae. aegypti*. It will be of interest to test the effect of overexpressing or silencing the expression of these DEGs on SINV replication in *Ae. aegypti* in order to further explain titer variability in this system and in other arbovirus-vector combinations.

## Figures and Tables

**Table 1:** DEGs with ≥10-fold expression changes relative to blood-fed ones that were observed in low titer midgut, high titer midgut, low titer carcass, and high titer carcass.

**Low Titer Midgut**				
**Gene ID**	**RefSeq**	**Gene Description**	**Fold Change**	**Adjusted *p*-Value**
AAEL021204	LOC110678065	uncharacterized	41.83931626	2.25 × 10^−26^
AAEL019786	LOC5570400	RYamide neuropeptide receptor	39.88676288	4.96 × 10^−36^
AAEL023555	LOC110678199	bypass of stop codon protein 1-like	33.39579316	9.73 × 10^−36^
AAEL004369	LOC5564633	alpha-glucosidase	32.01020047	1.60 × 10^−6^
AAEL022194	LOC110681519	cysteine and histidine-rich domain-containing protein-like	31.66204628	4.84 × 10^−6^
AAEL023070	LOC110678485	cytochrome b5-like	29.19310477	5.27 × 10^−5^
AAEL024968	LOC110676149	prisilkin-39-like	29.0463297	4.40 × 10^−22^
AAEL025215	LOC110676953	uncharacterized	27.60545214	1.44 × 10^−7^
AAEL023063	LOC110679464	uncharacterized	27.42324924	9.20 × 10^−17^
AAEL015345	LOC5570300	uncharacterized	26.78206758	1.32 × 10^−15^
AAEL022938	LOC5575293	Membrane trafficking protein emp24 domain-containing protein 7	25.61985948	2.62 × 10^−7^
AAEL027327	LOC110676057	uncharacterized	23.81667017	0.001830549
AAEL001364	LOC5570357	UDP-glucuronosyltransferase 3A1	23.77138085	2.04 × 10^12^
AAEL013570	LOC5578233	uncharacterized	23.76901847	1.80 × 10^−5^
AAEL008355	LOC5570460	uncharacterized	23.09585163	5.24 × 10^−20^
AAEL014170	LOC5563842	G8 domain-containing protein DDB_G0286311	22.51193091	9.46 × 10^−16^
AAEL027489	LOC5570061	uncharacterized	21.82318215	0.003321752
AAEL022803	LOC110680552	LMBR1 domain-containing protein 2 homolog	19.58182813	1.66 × 10^−8^
AAEL027016	LOC5576973	UDP-glucuronosyltransferase 2B15	−30.62089836	1.01 × 10^−5^
AAEL025019	LOC110680819	Ubiquinol-cytochrome c reductase complex assembly factor 6	−45.53775816	5.97 × 10^−19^
**High titer midgut**				
**Gene ID**	**RefSeq**	**Gene description**	**Fold change**	**Adjusted *p*-value**
AAEL021204	LOC110678065	uncharacterized	44.0209	8.18 × 10^−30^
AAEL019786	LOC5570400	RYamide neuropeptide receptor	42.24056	4.45 × 10^−41^
AAEL023555	LOC110678199	bypass of stop codon protein 1-like	35.27338	2.19 × 10^−40^
AAEL022938	LOC5575293	transmembrane emp24 domain-containing protein 7	34.89804	1.30 × 10^−15^
AAEL027489	LOC5570061	uncharacterized	33.20381	5.51 × 10^−7^
AAEL022194	LOC110681519	cysteine and histidine-rich domain-containing protein-like	31.87992	2.39 × 10^−6^
AAEL024968	LOC110676149	prisilkin-39-like	30.32939	1.80 × 10^−24^
AAEL022213	LOC110678859	26S proteasome non-ATPase regulatory subunit	30.2607	1.08 × 10^−5^
AAEL023063	LOC110679464	uncharacterized	30.07491	1.08 × 10^−20^
AAEL023070	LOC110678485	cytochrome b5-like	30.04727	1.28 × 10^−5^
AAEL015345	LOC5570300	uncharacterized	28.75877	2.42 × 10^−18^
AAEL001364	LOC5570357	UDP-glucuronosyltransferase 3A1	25.3656	1.47 × 10^−14^
AAEL008355	LOC5570460	uncharacterized	24.54677	5.08 × 10^−23^
AAEL013570	LOC5578233	uncharacterized	24.22035	6.72 × 10^−6^
AAEL014170	LOC5563842	G8 domain-containing protein DDB_G0286311	23.49	1.80 × 10^−17^
AAEL022803	LOC110680552	LMBR1 domain-containing protein 2 homolog	21.75694	4.38 × 10^−11^
AAEL020474	LOC5575291	methyltransferase-like protein 5	13.78589111	2.16 × 10^−13^
AAEL029015	LOC5574611	uncharacterized	10.44243	0.00678
AAEL007381	LOC5569108	uncharacterized	−11.0121	0.006332
AAEL019643	LOC5576254	mitochondrial ribosomal protein L53	−15.0348	0.003854
**Low titer carcass**				
**Gene ID**	**RefSeq**	**Gene description**	**Fold change**	**Adjusted *p*-value**
AAEL020237	LOC110681415	cyclin-dependent kinase 5 activator 1-like	19.32486515	6.13 × 10^−11^
AAEL017349	LOC23687769	heat shock 70 kDa protein cognate 3	18.49037534	1.43 × 10^−5^
AAEL019591	LOC5568644	uncharacterized	17.64663545	2.35 × 10^−11^
AAEL013721	LOC5578521	RISC-loading complex subunit tarbp2	17.10792675	7.77 × 10^−13^
AAEL011715	LOC5575241	non-structural maintenance of chromosomes element 1 homolog	16.4312446	3.92 × 10^−12^
AAEL021204	LOC110678065	uncharacterized	15.79915401	0.000187282
AAEL009330	LOC5571803	carbonic anhydrase II, putative	15.65841653	1.61 × 10^−12^
AAEL024640	LOC5574307	uncharacterized	15.41944881	5.09 × 10^−8^
AAEL024122	LOC110676237	uncharacterized	14.97321087	7.55 × 10^−9^
AAEL023803	LOC110675921	uncharacterized	14.7932861	3.76 × 10^−7^
AAEL021758	LOC110680549	uncharacterized	14.55831826	5.09 × 10^−8^
AAEL027718	LOC110676787	uncharacterized	14.40108737	6.85 × 10^−5^
AAEL007879	LOC5569726	uncharacterized	14.02614153	1.76 × 10^−6^
AAEL025296	LOC110679326	uncharacterized	13.4317571	0.000512544
AAEL009683	LOC5572259	uncharacterized	13.33276801	3.15 × 10^−5^
AAEL023555	LOC110678199	bypass of stop codon protein 1-like	13.24276866	3.89 × 10^−5^
AAEL003290	LOC5577738	cell wall protein DAN4 precursor, putative	13.05133731	2.55 × 10^−5^
AAEL023873	LOC5572852	uncharacterized	12.32245107	1.29 × 10^−5^
AAEL015285	LOC5566943	glutathione S-transferase T3	11.66563043	0.000184333
AAEL001364	LOC5570357	UDP-glucuronosyltransferase 3A1	−14.75718212	3.15 × 10^−5^
AAEL025361	LOC5571051	uncharacterized	−16.07641618	2.49 × 10^−6^
AAEL025907	LOC110673982	Pyruvate dehydrogenase [acetyl-transferring]-phosphatase 1, mitochondrial-like	−32.91331556	1.47 × 10^−8^
**High titer carcass**				
**Gene ID**	**RefSeq**	**Gene description**	**Fold change**	**Adjusted *p*-value**
AAEL019591	LOC5568644	uncharacterized	21.51946	1.80 × 10^−17^
AAEL000109	LOC5567790	Enolase-phosphatase E1	21.39589	8.86 × 10^−7^
AAEL011715	LOC5575241	non-structural maintenance of chromosomes element 1 homolog	20.57616	9.85 × 10^−20^
AAEL007879	LOC5569726	uncharacterized	20.47561	9.82 × 10^−16^
AAEL024122	LOC110676237	uncharacterized	20.41267	3.44 × 10^−18^
AAEL020237	LOC110681415	cyclin-dependent kinase 5 activator 1-like	20.23749	4.98 × 10^−12^
AAEL013721	LOC5578521	RISC-loading complex subunit tarbp2	20.23062	1.00 × 10^−18^
AAEL022938	LOC5575293	transmembrane emp24 domain-containing protein 7	20.16567	7.91 × 10^−6^
AAEL015285	LOC5566943	glutathione S-transferase T3	19.56361	4.18 × 10^−13^
AAEL017349	LOC23687769	heat shock 70 kDa protein cognate 3	19.55707	3.79 × 10^−6^
AAEL023873	LOC5572852	uncharacterized	18.83925	1.63 × 10^−15^
AAEL009330	LOC5571803	carbonic anhydrase 7	18.83826	2.29 × 10^−19^
AAEL024640	LOC5574307	uncharacterized	18.7741	1.19 × 10^−12^
AAEL027718	LOC110676787	uncharacterized	18.7525	3.55 × 10^−8^
AAEL009683	LOC5572259	uncharacterized	18.65438	2.60 × 10^−11^
AAEL021758	LOC110680549	uncharacterized	18.62961	9.39 × 10^−15^
AAEL000325	LOC5575187	probable cytochrome P450 313a2	16.52559	1.71 × 10^−6^
AAEL004369	LOC5564633	alpha-glucosidase	16.43245	0.000598
AAEL025296	LOC110679326	uncharacterized	14.10447	0.000246
AAEL019786	LOC5570400	RYamide neuropeptide receptor	13.91278	9.23 × 10^−5^
AAEL003290	LOC5577738	cell wall protein DAN4 precursor, putative	12.84058	2.17 × 10^−5^
AAEL023803	LOC110675921	uncharacterized	12.46491	3.38 × 10^−5^
AAEL025019	LOC110680819	uncharacterized protein C12orf73 homolog	10.66611	0.001682
AAEL009036	LOC5571393	uncharacterized	10.11032	0.000912
AAEL015345	LOC5570300	uncharacterized	−14.4716	4.16 × 10^−5^
AAEL001364	LOC5570357	UDP-glucuronosyltransferase 3A1	−15.1184	1.43 × 10^−5^
AAEL007381	LOC5569108	uncharacterized	−19.6795	0.000267

## Data Availability

Sequence data are available in the Sequence Read Archive (https://www.ncbi.nlm.nih.gov/sra) under BioProject ID PRJNA1000578.

## Supplementary Materials

The following supporting information can be downloaded at https://www.mdpi.com/article/10.3390/v16091487/s1: Figure S1: Validation of RNA-seq data analysis by RT-qPCR; Figure S2: GO term analysis of downregulated DEGs; Figure S3: GO term analysis of cellular compartments; Figure S4: The percentages of characterized versus uncharacterized DEGs; Table S1: Primers used for RT-qPCR; Table S2: Read counts and mapping results for each RNAseq sample post-QC; Supplementary File S1: Lists of DEGs exhibiting ≥ 2-fold changes in expression.
