# Supplementary material for: Differing Transcriptomic Responses in High Titer versus Low Titer Aedes aegypti Mosquitoes after Oral Infection with Sindbis Virus
Source: Viruses. 2024 Sep 19;16(9):1487. doi: 10.3390/v16091487 (PMC11437473; doi:10.3390/v16091487)
Supplement: Supplementary file 1 [file viruses-16-01487-s001.zip › Figure S1.pdf]

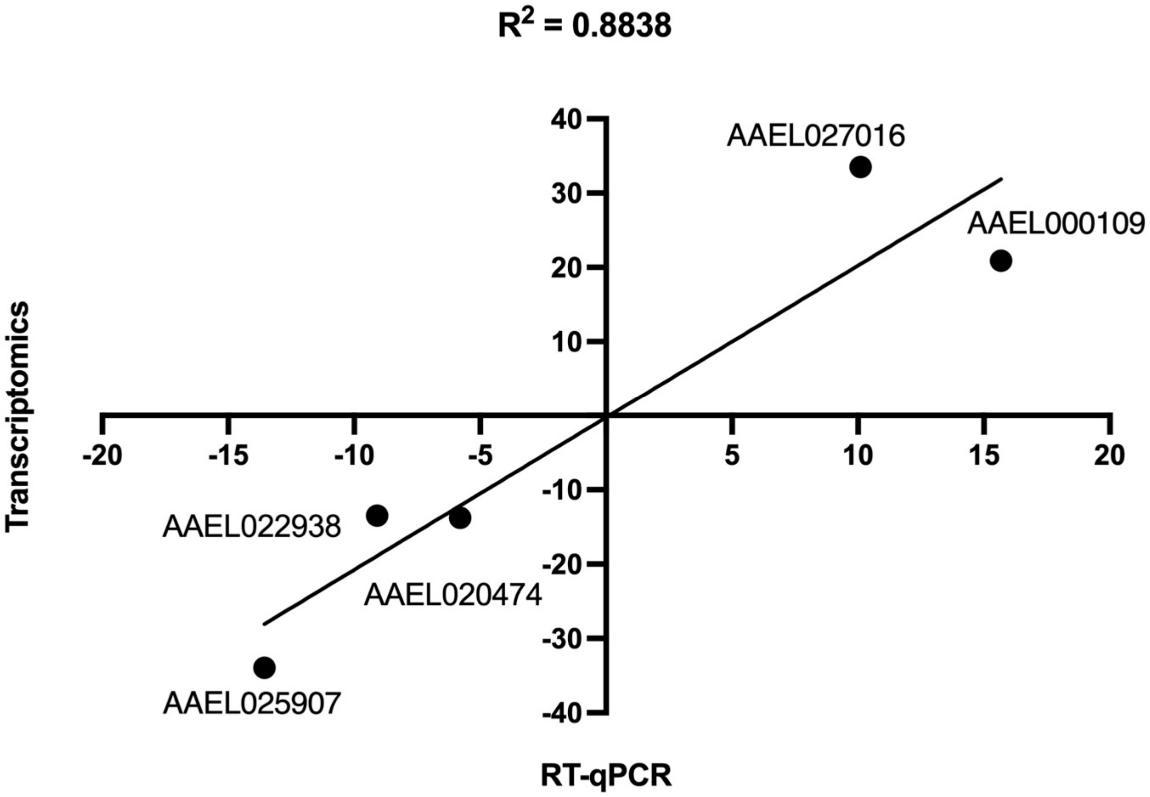

Figure S1: Validation of RNA-seq data analysis by RT-qPCR. The expression of five DEGs that exhibited read count differences of 10-fold or more compared to blood fed were validated by RT-qPCR.
