# Supplementary material for: Differing Transcriptomic Responses in High Titer versus Low Titer Aedes aegypti Mosquitoes after Oral Infection with Sindbis Virus
Source: Viruses. 2024 Sep 19;16(9):1487. doi: 10.3390/v16091487 (PMC11437473; doi:10.3390/v16091487)
Supplement: Supplementary file 1 [file viruses-16-01487-s001.zip › Figure S2.pdf]

A

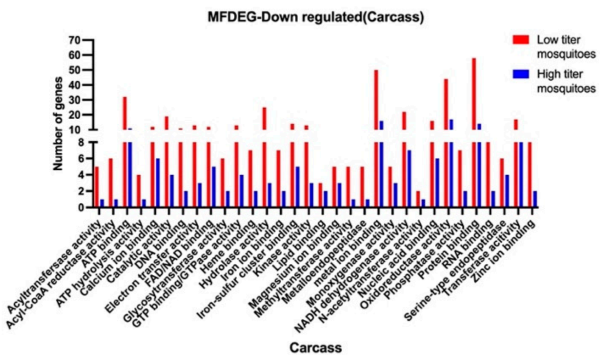

B

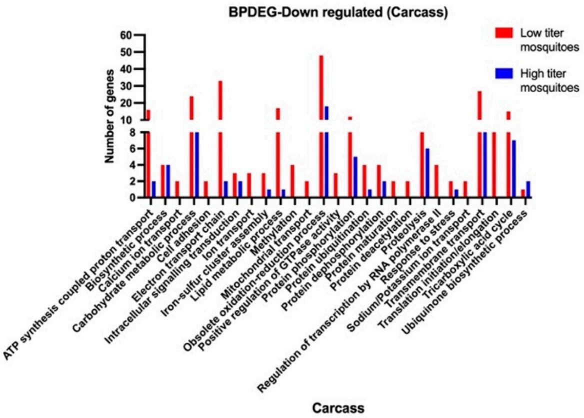

Figure S2: GO term analysis of downregulated DEGs in carcasses of low titer and high titer mosquitoes showing (A) molecular function (B) biological process in carcasses. The results for downregulated DEGs in midguts are not shown due to the majority of the genes being uncharacterized.
