# Supplementary material for: Differing Transcriptomic Responses in High Titer versus Low Titer Aedes aegypti Mosquitoes after Oral Infection with Sindbis Virus
Source: Viruses. 2024 Sep 19;16(9):1487. doi: 10.3390/v16091487 (PMC11437473; doi:10.3390/v16091487)
Supplement: Supplementary file 1 [file viruses-16-01487-s001.zip › Figure S3.pdf]

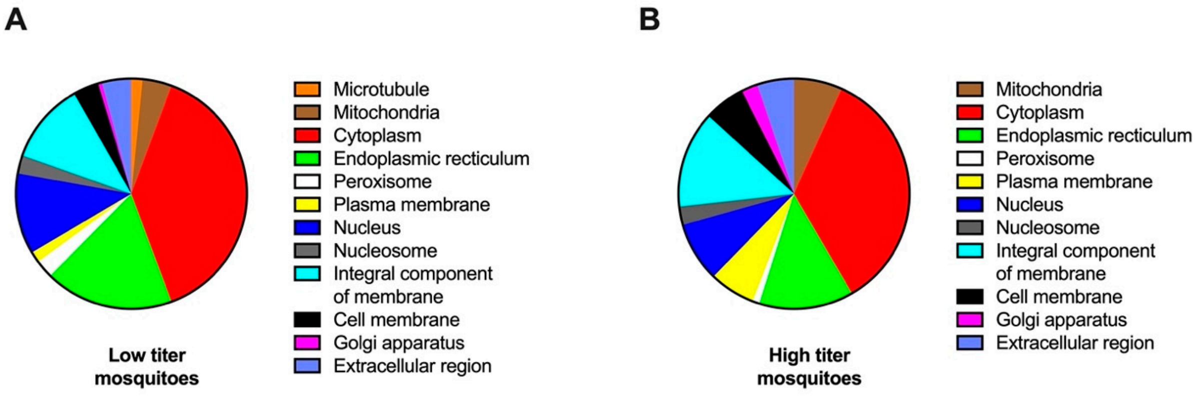

Figure S3: GO term analysis of the cellular compartments where the products of DEGs are predicted to function in (A) low titer and (B) high titer mosquitoes.
