# Supplementary material for: Differing Transcriptomic Responses in High Titer versus Low Titer Aedes aegypti Mosquitoes after Oral Infection with Sindbis Virus
Source: Viruses. 2024 Sep 19;16(9):1487. doi: 10.3390/v16091487 (PMC11437473; doi:10.3390/v16091487)
Supplement: Supplementary file 1 [file viruses-16-01487-s001.zip › Supplementary File S1.pdf]

Supplementary File S1. Excel spreadsheet listing the DEGs exhibiting ≥2-fold changes in expression compared to blood fed controls
