# Supplementary material for: Differing Transcriptomic Responses in High Titer versus Low Titer Aedes aegypti Mosquitoes after Oral Infection with Sindbis Virus
Source: Viruses. 2024 Sep 19;16(9):1487. doi: 10.3390/v16091487 (PMC11437473; doi:10.3390/v16091487)
Supplement: Supplementary file 1 [file viruses-16-01487-s001.zip › Table S1.pdf]

Table S1. Primers used for RT-qPCR

Primer name	Gene ID	Primer sequence
Methyltransferase F	AAEL020474	AGCCAAGCTCAATCGAACCA
Methyltransferase R		CGAACCTGGTCCCGAACTG
UDP-glucuronosyltransferase F	AAEL027016	AGTGAATGGGCAGTCTAAAGC
UDP-glucuronosyltransferase R		GACTTGGACTAGGAACCGTCA
Pyruvate dehydrogenase F	AAEL025907	AGCTGTCGGGTCGGGTG
Pyruvate dehydrogenase R		ACCGCACTTCACTTCAGTACC
Transmembrane emp24 F	AAEL022938	CGCGTGTACGCGTATATCAAA
Transmembrane emp24 R		ACGATGCCTCCGCAATACTT
Enolase-phosphatase F	AAEL000109	GACCATGTGAAAAATGAACAATGTG
Enolase-phosphatase R		TGTGCTTTTGGAAAGTCGGTA
RPS7 F	AAEL009496	CTTTGATGTGCGAGTGAACAC
RPS7 R		CATCTCCAACTCCAGGATAGC
