# Supplementary material for: Differing Transcriptomic Responses in High Titer versus Low Titer Aedes aegypti Mosquitoes after Oral Infection with Sindbis Virus
Source: Viruses. 2024 Sep 19;16(9):1487. doi: 10.3390/v16091487 (PMC11437473; doi:10.3390/v16091487)
Supplement: Supplementary file 1 [file viruses-16-01487-s001.zip › Table S2.pdf]

Table S2. Read counts and mapping results for each RNAseq sample post-QC

Sample	Read count post-QC	Percent reads mapped to *Ae. aegypti*
BFM1	13,656,552	90%
BFM2	17,076,302	90%
BFM8	22,125,783	91%
BFC1	15,401,269	90%
BFC2	14,579,719	89%
BFC8	17,625,035	89%
LTM95	17,091,375	87%
LTM175	21,100,739	89%
LTM245	14,507,985	79%
LTM247	15,027,824	83%
LTM259	20,279,448	85%
LTM265	10,830,843	82%
LTC95	6,485,800	27%
LTC175	10,976,355	87%
LTC245	5,550,358	46%
LTC247	8,574,219	43%
LTC259	7,620,692	42%
LTC265	7,305,382	44%
HTM7	11,636,371	75%
HTM27	10,706,186	82%
HTM170	18,124,704	84%

HTM180	17,927,873	83%
HTM201	16,028,397	80%
HTM237	12,349,097	77%
HTC7	7,382,396	27%
HTC27	9,838,361	61%
HTC170	5,674,470	33%
HTC180	5,222,201	27%
HTC201	4,015,663	21%
HTC237	6,992,814	45%
